# Nuclear Factor κ-B Is Activated in the Pulmonary Vessels of Patients with End-Stage Idiopathic Pulmonary Arterial Hypertension

**DOI:** 10.1371/journal.pone.0075415

**Published:** 2013-10-04

**Authors:** Laura C. Price, Gaetano Caramori, Frederic Perros, Chao Meng, Natalia Gambaryan, Peter Dorfmuller, David Montani, Paolo Casolari, Jie Zhu, Konstantinos Dimopoulos, Dongmin Shao, Barbara Girerd, Sharon Mumby, Alastair Proudfoot, Mark Griffiths, Alberto Papi, Marc Humbert, Ian M. Adcock, S. John Wort

**Affiliations:** 1 Unit of Critical Care, Royal Brompton Hospital, National Heart and Lung Institute, Faculty of Medicine, Imperial College London, London, United Kingdom; 2 Lung Pathology, Imperial College London, Royal Brompton Hospital, London, United Kingdom; 3 Cell and Molecular Biology, Airways Disease Section, National Heart and Lung Institute, Faculty of Medicine, Imperial College London, London, United Kingdom; 4 Univ. Paris-Sud, Le Kremlin-Bicêtre, France; 5 Institut National de la Santé et de la Recherche Médicale UMR_S 999, LabEx LERMIT, Centre Chirurgical Marie Lannelongue, Le Plessis Robinson, France; 6 Assistance publique–Hôpitaux de Paris, Service de Pneumologie, DHU Thorax Innovation, Hôpital Bicêtre, Le Kremlin-Bicêtre, France; 7 Pathology Department, Centre Chirurgical Marie Lannelongue, Le Plessis-Robinson, France; 8 Section of Respiratory Diseases, Centro Interdipartimentale per lo Studio delle Malattie Infiammatorie delle Vie Aeree e Patologie Fumo-correlate, University of Ferrara, Ferrara, Italy; University of Illinois College of Medicine, United States of America

## Abstract

**Objectives:**

To assess activation of the inflammatory transcription factor NF-kappa B (NF-κB) in human idiopathic pulmonary arterial hypertension (PAH).

**Background:**

Idiopathic PAH is a severe progressive disease characterized by pulmonary vascular remodeling and excessive proliferation of vascular cells. Increasing evidence indicates that inflammation is important in disease pathophysiology.

**Methods:**

NF-κB-p65 and CD68, CD20 and CD45 were measured by immunohistochemistry and confocal microscopy on lung specimens from patients with idiopathic PAH (n = 12) and controls undergoing lung surgery (n = 14). Clinical data were recorded for all patients including invasive pulmonary hemodynamics for the PAH patients. Immunohistochemical images were analyzed by blinded observers to include standard pulmonary vascular morphometry; absolute macrophage counts/mm^2^ and p65-positivity (p65+) using composite images and image-analysis software; and cytoplasmic:nuclear p65+ of individual pulmonary arterial endothelial and smooth muscle cells (PASMC) in 10–20 pulmonary arteries or arterioles per subject. The expression of ET-1 and CCL5 (RANTES) in whole lung was determined by RT-qPCR.

**Results:**

Macrophage numbers were increased in idiopathic PAH versus controls (49.0±4.5 vs. 7.95±1.9 macrophages/100 mm^2^, p<0.0001): these macrophages demonstrated more nuclear p65+ than in macrophages from controls (16.9±2.49 vs. 3.5±1.25%, p<0.001). An increase in p65+ was also seen in perivascular lymphocytes in patients with PAH. Furthermore, NF-κB activation was increased in pulmonary arterial endothelial cells (62.3±2.9 vs. 14.4±3.8, p<0.0001) and PASMC (22.6±2.3 vs. 11.2±2.0, p<0.001) in patients with PAH versus controls, with similar findings in arterioles. Gene expression of both ET-1 mRNA ((0.213±0.069 vs. 1.06±0.23, p<0.01) and CCL5 (RANTES) (0.16±0.045 vs. 0.26±0.039, p<0.05) was increased in whole lung homogenates from patients with PAH.

**Conclusions:**

NF-κB is activated in pulmonary macrophages, lymphocytes, endothelial and PASMC in patients with end-stage idiopathic PAH. Future research should determine whether NF-κB activation is a driver or bystander of pulmonary vascular inflammation and if the former, its potential role as a therapeutic target.

## Introduction

Pulmonary arterial hypertension (PAH) is a severe disease characterized by excessive proliferation of vascular cells leading to progressive pulmonary vascular remodeling, right ventricular failure and premature death [1], [2]. The role of inflammation is increasingly recognized in the pathogenesis of several PAH sub-types (e.g. connective-tissue disease and HIV-associated PAH) as well as idiopathic disease [3]–[6]. Examination of diseased lung tissue reveals perivascular inflammatory cell infiltrates (including lymphocytes, monocytes, macrophages, dendritic cells, mast cells and bone-marrow-derived progenitor cells) [7]–[11]. Furthermore, cytokines and chemokines including interleukin (IL-1)-β, IL-6, CXCL8/IL-8, CCL2 (MCP-1), CCL5 (RANTES) and tumor necrosis factor (TNF)-α are elevated in plasma or tissue of patients [12]–[17], some of which in addition are predictive of worse outcomes [15], [16]. Potential immune activators in PAH include viral and parasitic infections, oxidative stress and dysfunction of the transforming growth factor-β (TGF-β)/BMPRII signaling cascade [5]. Upregulation of pro-inflammatory genes in response to environmental triggers is coordinated by transcription factors, of which nuclear factor-kappa B (NF-κB) is central [18]. NF-κB is ubiquitously expressed with the most common NF-κB complex consisting of heterodimeric p50 and p65 (RelA) subunits. Under normal conditions, this complex remains quiescent in the cytoplasm bound to an inhibitory protein, IκBα. Upon activation, IκBα is phosphorylated and degraded; the heterodimer translocates into the nucleus and increases expression of pro-inflammatory genes including ET-1 and CCL5, as well as those involved in promoting vascular cell growth and angiogenesis [19], [20].

Activation of NF-κB is a feature of many chronic inflammatory conditions including asthma [21], chronic obstructive pulmonary disease [22], and rheumatoid arthritis [23] as well as in (non-lymphoid) malignancies [24]. In asthma and COPD, activation of NF-κB correlates with disease severity, suggesting a direct role in pathogenesis [21], [22]. In atherosclerosis, NF-κB activation is seen within macrophages, endothelial and vascular smooth muscle cells within atherosclerotic plaques [25]: the degree of activation is positively associated with symptoms of unstable angina [26], [27].

Several lines of evidence suggest that NF-κB may play a role in PAH. Cell-based studies of normal pulmonary arterial smooth muscle cells (PASMC) show that NF-κB is activated by inflammatory or proliferative stimuli [28], [29], and that it mediates cytokine-induced release of endothelin-1 [30]. NF-κB is also activated in the monocrotaline model of pulmonary hypertension (PH) in rats where its blockade ameliorates PH [31], [32]. Increased NF-κB activation has been demonstrated in alveolar macrophages obtained from bronchoalveolar lavage from patients with PAH [33], but has not been previously been examined in lung tissue from these patients.

We therefore performed a detailed immunohistochemical assessment of lung stained for the p65 subunit of NF-κB [34], as well as specific inflammatory cell markers, hypothesizing that NF-κB activation in both inflammatory and pulmonary vascular cells was a feature of patients with end stage idiopathic PAH. In addition we assessed mRNA for the NF-κB-regulated genes ET-1 and CCL5 in lung tissue from PAH patients compared to control subjects.

## Methods

### Subjects

Control specimens (n = 14) were obtained from lung resected during lobectomy for a solitary pulmonary nodule; for patients with idiopathic PAH (n = 12), specimens were obtained following lung transplantation. Demographics, co-morbidities, drug history and inflammatory markers were recorded, as well as clinical, haemodynamic status and PAH therapies prior to transplantation. Detailed operating procedures for written consent, collection, documentation and processing of frozen lung samples from patients with PAH and controls were developed for the constitution of a tissue bank associated with the National PAH Referral Centre and for this purpose approval was obtained by the French Ministry of Education and Research, as well as by the Regional Health Agency Ile-de-France, by the Local Ethics Committee of University Hospital of Ferrara, Italy, and the Review board of the Biomedical Research Unit, Royal Brompton Hospital, London, UK. Written consent was obtained from all participants prior to the study and all consent forms were collected at all centres and documents kept in a file for future reference.

### Lung Tissue Processing

Two to four randomly selected blocks of lung tissue (size 2×2.5 cm) were taken from the sub-pleural parenchyma of the lobe obtained at the time of surgery, avoiding areas involved by tumor in controls. Fresh lung tissue was gently inflated through the bronchi using an automated formalin-pump (Microm, France) and eventually immersed in buffered formalin for 24 hours. All lobes were sampled. For each case, 30–40 samples were embedded in paraffin and used for histological analysis using 4 µm thick sections.

### Immunoperoxidase Staining of Lung Sections

Sections were dewaxed and rehydrated as previously described [35], followed by heat-mediated antigenic retrieval using a pH 6.0 citrate buffer. Endogenous peroxidase activity was quenched by incubation of the slides in 3% hydrogen peroxide for 30 minutes; cell membranes were permeabilised by 0.1% saponin in PBS, and non-specific labelling blocked by 5% normal horse serum in PBS for 20 minutes, at room temperature (as for all the following steps). After washing in PBS, sections were incubated for 1 hour with mouse anti-human CD68 (Dako) at a 1∶100 dilution (160 µg/ml). For negative control slides, normal mouse non-specific immunoglobulins (Santa Cruz Biotechnology) were used at the same protein concentration as the primary antibody. After repeated PBS washing, sections were incubated with anti-mouse biotinylated antibody (Vector ABC Kit, Vector Laboratories) for 30 minutes. Sections were then incubated with ABC reagent (Vector ABC Kit, Vector Laboratories) for 30 minutes and stained with chromogen-fast diaminobenzidine (DAB) for 1–5 minutes. Selected slides were incubated with anti-CD20 (Diagnostic BioSystems, Pleasanton, USA) anti-CD45 (Thermo Fisher Scientific, Cheshire, UK).

For CD68-p65 double staining, the process was repeated again on the same slides for staining p65 using a rabbit anti human p65 (Santa Cruz Biotechnology) at a dilution of 1∶50 (200 µg/ml) or a normal rabbit non-specific immunoglobulins (Santa Cruz Biotechnology) at the same protein concentration as the primary antibody for negative control. Finally, the slides were stained with chromogen fast red for 10–20 minutes, counterstained in haematoxylin and mounted on aqueous mounting medium.

### Morphometric Analysis

Microscopy was performed in the Facility for Imaging by Light Microscopy (FILM) at Imperial College London, and at the Royal Brompton Hospital, London UK. Absolute numbers of CD68+ macrophages/mm^2^ were counted in at least 20 fields of view using stitched images obtained using the 10× objective on the Zeiss Axiovert 200 inverted microscope, with area measured using Improvision Volocity acquisition software. For pulmonary arterial endothelial cells (EC) and PASMC, individual cells in 10–20 pulmonary arteries (external diameter [ED] 100–500 µm) per subject were analysed under high-power magnification (x400). Additional standard morphometric data included pulmonary arterial ED (µm) and percentage smooth muscle medial thickness (single medial thickness/ED x100). p65-positive (p65+) cells were quantified for overall p65+ staining by two independent blinded observers (LCP and JZ). Arterioles (ED<100 µm) were also evaluated for overall p65+ EC and p65+ PASMC. Further to overall p65+ cellular positivity, it was also possible to perform cytoplasmic and/or nuclear p65+ assessment for CD68+ cells and EC, although not for PASMC as this whole cell including the nucleus was not always seen. The mean, intra-observer and inter-observer variability coefficients for counting were less than 10%.

### Immunofluorescence

Staining was performed on 5 µm acetone-fixed frozen sections. The sections were incubated for 1 h in 10% human serum to block non-specific protein-protein interactions. The sections were then incubated with primary anti-NF-κB p65 (acetyl K310) antibody (Abcam, ab52175) at a 1/100 dilution overnight at 4°C. AlexaFluor488-conjugated goat anti-rabbit secondary antibody (Invitrogen) was used to reveal the staining. DAPI was used to stain the cell nuclei.

### RNA Extraction and Real Time qRT-PCR

For each subject, 30 mg of human lung tissue was homogenized in RLT buffer and total RNA was extracted using RNeasy® mini kit (Qiagen). Total RNA (2 µg) was transcribed to cDNA using L-AMV reverse transcriptase (Invitrogen) according to the manufacturer’s instructions. Real-time quantitative PCR was performed using SYBR green master mix on a Corbett Rotor-Gene 6000 (Qiagen). The relative expression of target genes was quantified using the ΔΔCt method normalized to GAPDH [36]. The QuantiTech® Primer assay for ET-1, RANTES and GAPDH were purchased from Qiagen, details as follow: EDN-1(ET-1), QT00088235; CCL-5 (RANTES), QT00090083; GADPH, 01192646.

### Statistical Analysis

The sample size (n = 12 per group) was selected with the scope of detecting a difference in total CD68+ cell (macrophage) count between the two groups of 1.5 standard deviations at a significance level of 0.01 and power of 80%, based on data from previous studies [35]. Numeric data were expressed as mean ± standard error of the mean (SEM) or median (interquartile range). Comparisons in continuous variable between groups were performed using Student’s t test for normally distributed data and Mann-Whitney U test for non-normally distributed variables. Linear regression was used for assessing the relation between continuous variables. A p-value <0.05 was considered indicative of statistical significance. GraphPad Prism 5.0 (La Jolla, California) and R version 2.13.0 were used for statistical analyses and the generation of plots.

## Results

### Clinical Findings

Both groups had a female predominance. As expected, patients with idiopathic PAH were younger than patients undergoing lung cancer surgery. None were current smokers. At the time of preoperative assessment, controls had no evidence of airway disease on lung function and none were taking corticosteroid-based therapy, including inhalers. There was no difference in total or differential peripheral blood white cell counts (Table 1). Most PAH patients had severely increased pulmonary arterial pressures and were in NYHA functional class III or IV at their pre-transplantation assessment. Two patients had known *BMPR2* mutations (Table 2).

**Table 1:** Demographic and biochemical characteristics of study subjects.

	Controls (n = 14)	PAH patients (n = 12)
Age (years)*	68 (2)	36 (10)***
M/F	4/10	5/7
Smoking history (NS/FS)	11/3	0
CRP (mg/L)	NA	<10 (<10–47)
Peripheral WCC (absolute)		
Total count (x10^−9^/L)	6.93 (6.25–9.19)	7.90 (5.80–9.25)
Neutrophils (x10^−9^/L)	5.00 (3.79–7.65)	5.70 (3.80–7.05)
Lymphocytes (x10^−9^/L)	1.63 (0.85–1.91)	1.40 (0.80–1.55)
Monocytes (x10^−9^/L)	0.50 (0.40–0.59)	0.50 (0.40–0.65)
Eosinophils (x10^−9^/L)	0.10 (0.04–0.14)	0.20 (0.10–0.25)
Basophils (x10^−9^/L)	0.00 (0.0–0.025)	0.00 (0.0–0.01)

* At pre-transplantation assessment (for all measurements).

*** p<0.0001 between groups.

Values are mean (SEM) or median (IQR).

Abbreviations: M male, F female, NS non-smoker, FS former smoker, CRP C-reactive protein, WCC white cell count, NA data not available.

**Table 2:** Clinical characteristics of idiopathic PAH patients.

	PAH patients (n = 12)
Age at diagnosis (years)	31 (3)
Years from diagnosis to LTx	4.8 (1.6)
NYHA functional class (III/IV, n)*	5/7
6 MWD (m) (n = 6)	391 (33)
BNP (pg/ml)	259 (86.1)
*BMPR2* mutation status (mutant/wild type) (n = 10)	2/8
RAP (mmHg)	8 (1.7)
mPAP (mmHg)	62 (15.4)
PCWP (mmHg) (n = 10)	8 (1.6)
CO L/min	4.80 (0.70)
CI L/min/kg/m^2^	2.80 (0.31)
PVRI (n = 10) (Wood units-m^2^)	21.1 (2.7)
PAH therapies (n)	Epoprostenol (12), bosentan (12), sildenafil (7)

* At last assessment prior to lung transplantation (for all measurements).

Values are mean (SEM).

Abbreviations: LTx lung transplantation, NYHA New York Heart Association, BNP brain natriuretic peptide, BMPR2 bone morphogenetic protein receptor type 2, RAP right atrial pressure, mPAP mean pulmonary artery pressure, PCWP pulmonary capillary wedge pressure, CO cardiac output, CI cardiac index, PVRI pulmonary vascular resistance index.

### Pulmonary Artery Morphometric Assessment

**Figure 1:**
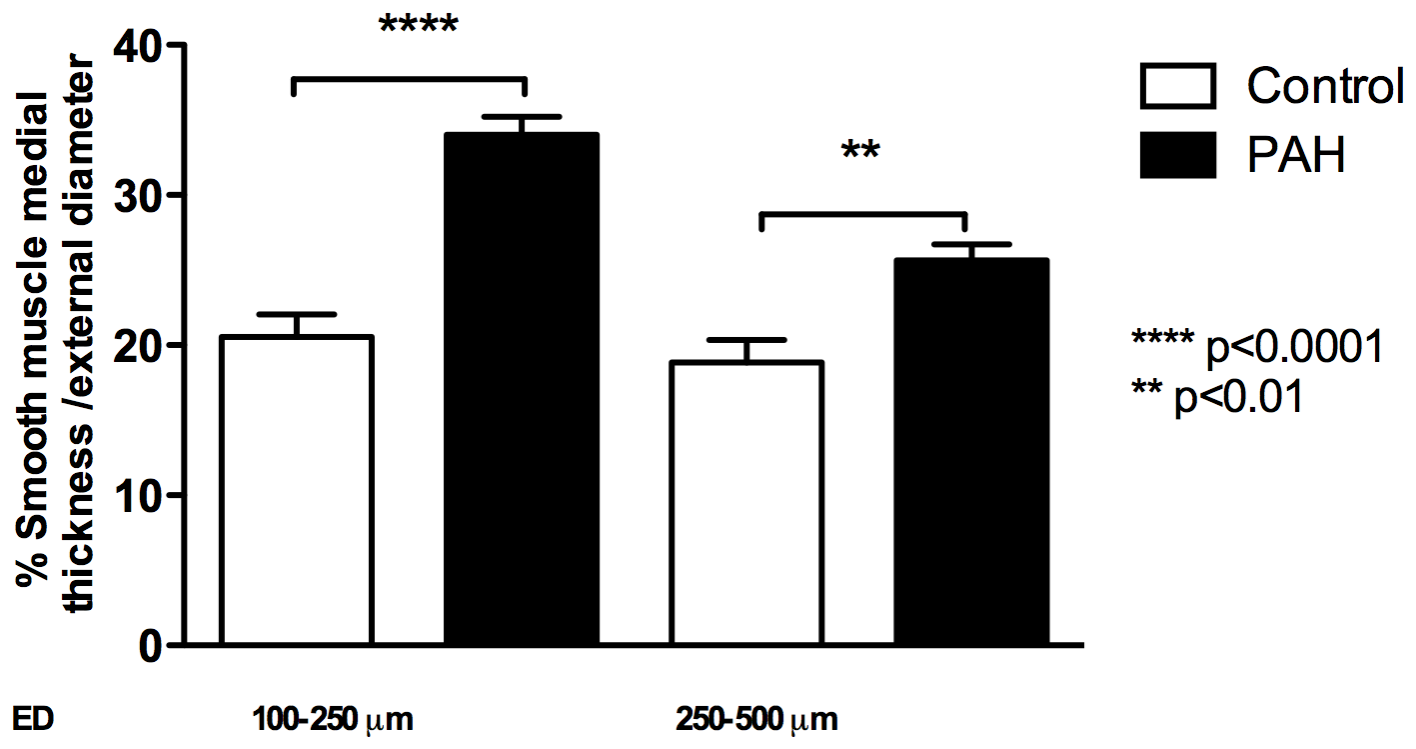
Pulmonary arterial smooth muscle medial thickness. Percentage medial thickness increased in pulmonary arterial hypertension (PAH) compared to controls from 20.5±1.5 to 34.0±1.2 (***p<0.001) in vessels 100–250 µm external diameter (ED) and from 18.9±1.5 to 25.6±1.08 (**p<0.01) in vessels 250–500 µm ED. Results expressed as mean ± SEM of 12 PAH and 14 control samples.

PAH patients had significantly higher smooth muscle medial thickness in vessels with 100–250 µm ED (34.0±1.2% versus 20.5±1.5% in controls, p<0.0001) and in vessels with 250–500 µm ED (25.6±1.1% versus 18.86±1.5% in controls, p<0.01, Figure 1). There were no significant correlations seen between vessel medial thickness and clinical and hemodynamic parameters (Figure S1 in File S1).

### Absolute Counts of Parenchymal Macrophages

**Figure 2:**
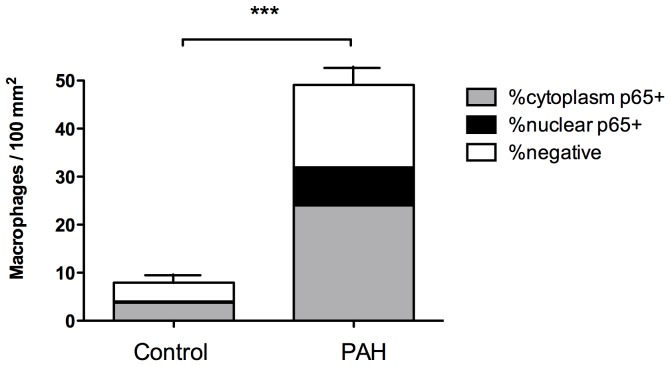
Macrophage numbers in idiopathic pulmonary arterial hypertension. Numbers of macrophages per 100^2^ of lung parenchyma were increased in pulmonary arterial hypertension (PAH) vs. controls (49.0±4.5 vs. 7.95±1.9/100 mm^2^, ***p<0.001). Additionally, p65+ macrophages were increased in PAH compared with controls (52.4±2.15% vs. 71.6±2.98%, ***p<0.001) and in the nucleus in PAH compared to controls (3.5±1.3% vs. 16.9±2.5%, ***p<0.001). Results are expressed as mean ± SEM for 12 PAH and 14 control subjects.

The total CD68+ cell (macrophage) count was higher in PAH patients (49.0±4.5 versus 7.95±1.9 macrophages/100 mm^2^ in controls, p<0.0001) (Figure 2). Within the PAH group, a positive correlation was seen with brain natriuretic peptide (BNP) (Pearson r = 0.75, p = 0.01), and a negative correlation with central venous oxygen saturations (SvO_2_) (r = −0.83, p = 0.02), which are markers of poor cardiac function and cardiac output respectively. No significant correlation was seen with any other hemodynamic index or markers of disease severity. (Figure S2 in File S1).

### p65 Immunohistochemistry in Parenchymal Macrophages

**Figure 3:**
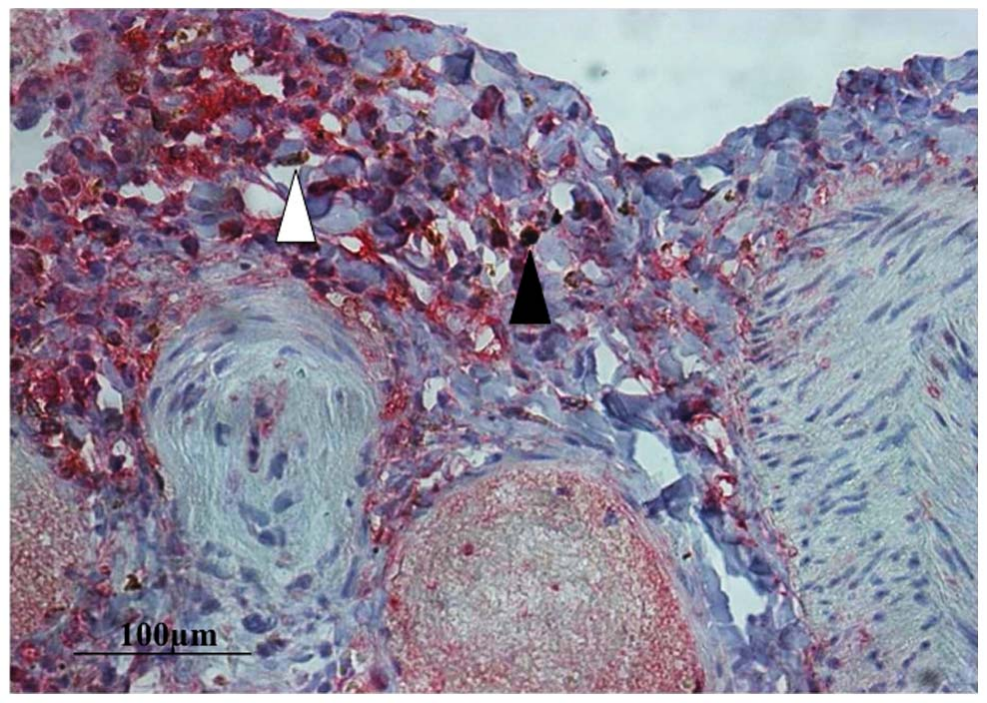
NF-κB staining in perivascular macrophages in lung parenchyma a patient with idiopathic pulmonary arterial hypertension. Macrophages (CD68+ cells, brown) are seen surrounding a diseased pulmonary artery in a post-transplantation lung specimen from a patient with idiopathic pulmonary arterial hypertension (PAH). Some of these cells exhibit nuclear p65-positive staining (pink staining, black arrow) i.e. show evidence of NF-κB activation; others demonstrate p65-negative staining (CD-68 brown only with no pink counter-staining, white arrow). In addition, small mononuclear cells (lymphocytes) stain avidly for p65. Magnification x200.

**Figure 4:**
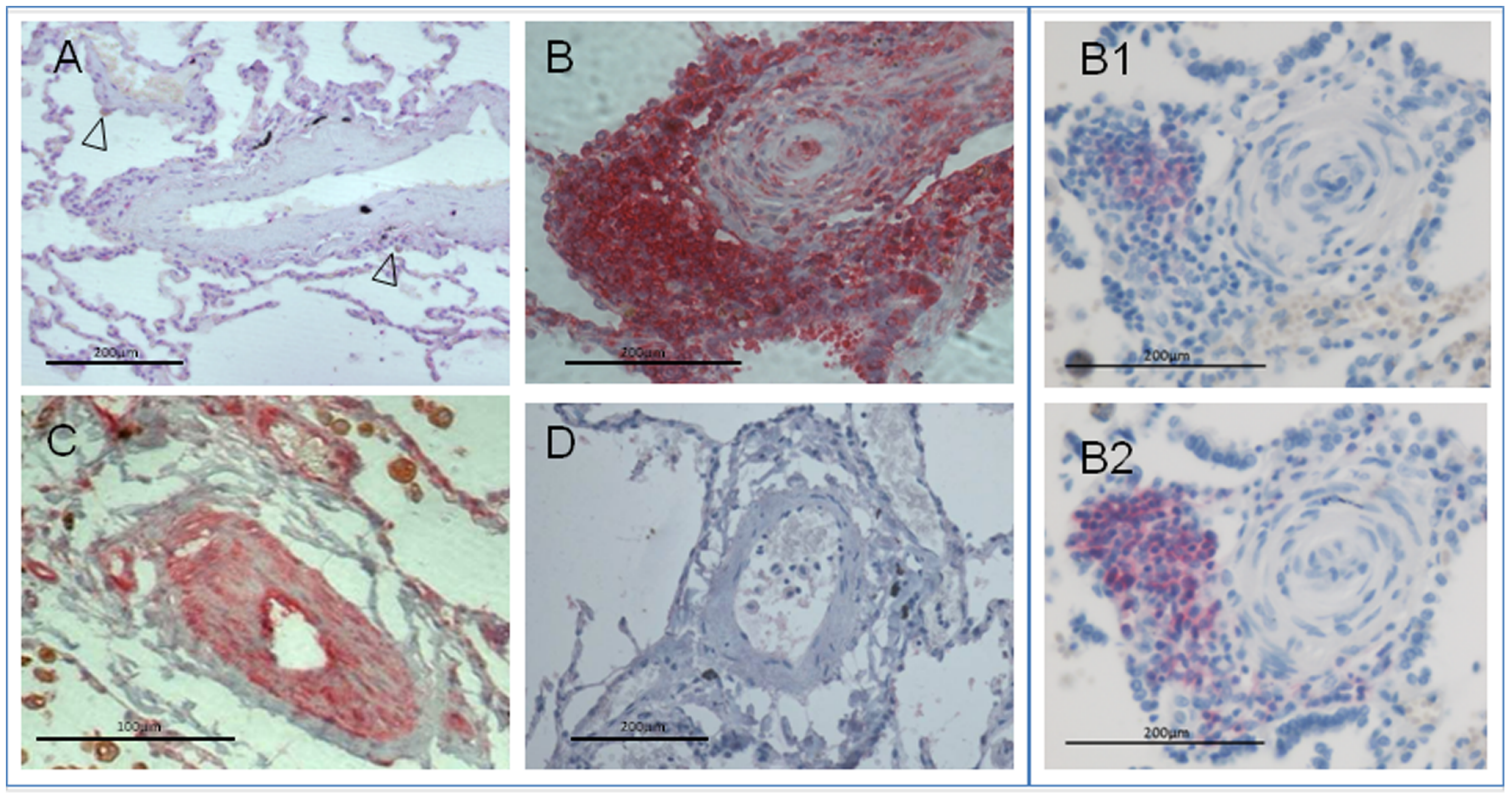
NF-κB staining in a control subject and in patients with idiopathic pulmonary arterial hypertension. (A) Control pulmonary artery showing p65- staining in sparse macrophages (arrows) and p65- vascular cells (x200). (B) Occluded pulmonary artery in a patient with idiopathic pulmonary arterial hypertension (PAH) showing intense p65 staining in pulmonary vascular cells and perivascular inflammatory cells, with further serial sections showing these cells also stain positively for B lymphocytes (CD20, Figure B1) and T lymphocytes (CD45, Figure B2). (C) High expression of p65 in endothelial cells (EC) and in PASMC nuclei in a pulmonary arteriole (x400). (D) Negative antibody-control in a non-PAH control (normal mouse IgG+normal rabbit IgG) (x200). Images representative of 12 iPAH and 14 control subjects.

Examination of lung parenchyma showed that the number of p65+ macrophages was higher in PAH: 71.6±2.98% compared to 52.4±2.15% in controls, p<0.001. When assessing p65+ macrophages, the number of nuclear p65+ cells was also higher in PAH patients: 16.9±2.5% compared 3.5±1.25% in controls, p<0.001 (Figures 2, 3, 4). Despite the increase in macrophage p65+, within the PAH group, no correlation was seen with any clinical or haemodynamic parameters (Figure S3 in File S1), or macrophage p65+ with vessel medial thickness (not shown).

### p65 Immunohistochemistry in Pulmonary Arterial Vascular Cells

**Figure 5:**
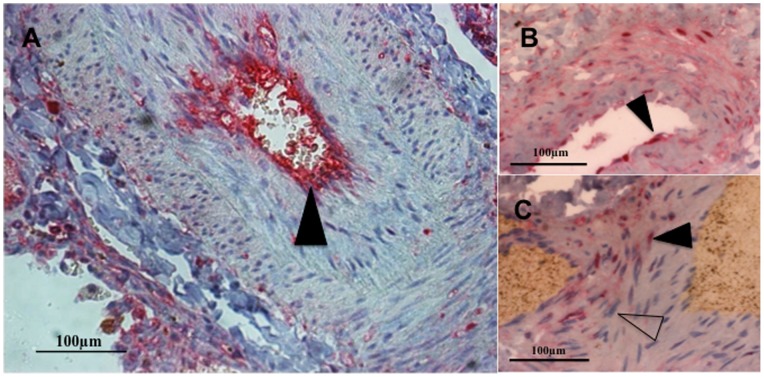
NF-κB p65 staining in pulmonary vascular cells in lung parenchyma a patient with idiopathic pulmonary arterial hypertension. Light microscopy demonstrating immunohistochemical p65+ (pink)-staining in vascular cells in diseased pulmonary arteries in lung sections from pulmonary arterial hypertension (PAH) subjects. Pulmonary arterial endothelial cells show intense staining for p65+ as indicated with a black arrow (A and B). Pulmonary arterial smooth muscle cells (PASMC) stain positive for nuclear p65+ (black arrow) and p65-stain negative (unfilled arrow) (C). Results representative of those from 12 PAH subjects. Magnification x200.

**Figure 6:**
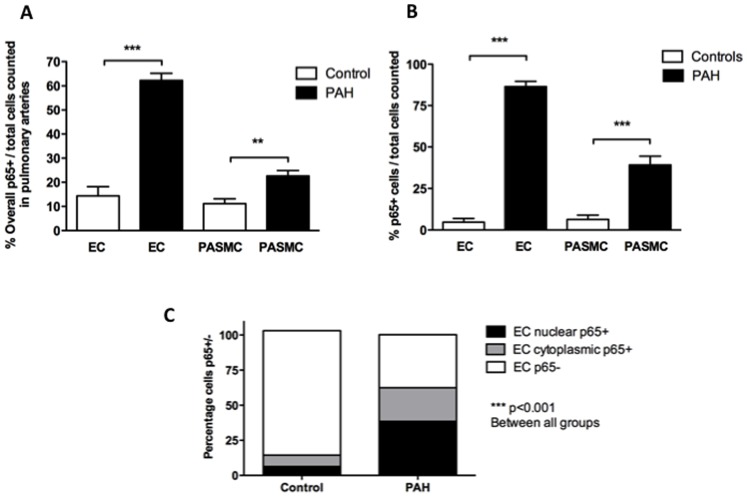
Quantification of NF-κB p65 staining in pulmonary vascular cells. (A) Overall p65+ was increased in pulmonary arterial endothelial cells (EC) (62.3±2.9 vs. 14.4±3.8%, ***p<0.001), pulmonary arterial smooth muscle cells (PASMC) (22.6±2.3 vs. 11.2±2.0%, ***p<0.001) in PAH versus controls and (B) within pulmonary *arterioles* in both EC (86.4±3.28 vs. 4.68±2.29%, p<0.0001) and PASMC (39.2±5.29 vs. 6.39±2.61%, ***p<0.001). (C) In pulmonary endothelial cells, percentage p65+ was increased in pulmonary arterial EC in PAH versus control subjects following subdivision into cytoplasmic p65+ (23.7±1.6 vs. 8.0±2.2%, PAH, ***p<0.001) and nuclear p65+ (38.5±2.1 vs. 6.4±1.6%, ***p<0.001). Results represent mean ± SEM of 12 PAH and 14 control samples.

In pulmonary arteries (ED 100–500 µm) a markedly higher number of p65+ ECs was observed in PAH (62.3±2.9 versus 14.4±3.8%, p<0.0001). A significant difference was also observed in PASMC (22.6±2.3 in PAH versus 11.2±2.0% in controls, p<0.001) (Figures 4–5 and 6a). A similar pattern was seen in pulmonary arterioles, where the number of p65+ EC (86.4±3.28 versus 4.7±2.3 in controls) and PASMC (39.2±5.3 in PAH versus 6.39±2.61 in controls) was significant higher in PAH (p<0.0001 for both, Figure 6b). Further analysis of pulmonary arterial EC showed higher numbers of overall p65+ (23.7±1.6 in PAH versus 8.0±2.2% in controls, p<0.0001) and nuclear p65+ cells (38.5±2.1 in PAH versus 6.4±1.6 in controls, p<0.0001, Figure 6c). There was no significant correlation seen with EC or PASMC p65+ and any clinical or haemodynamic variables (Figure S4 and S5 in File S1). In addition, no significant correlation was seen within groups between for EC and PASMC p65+ and vessel medial thickness (not shown).

### p65 Immunofluorescence Staining in Pulmonary Vascular Cells

**Figure 7:**
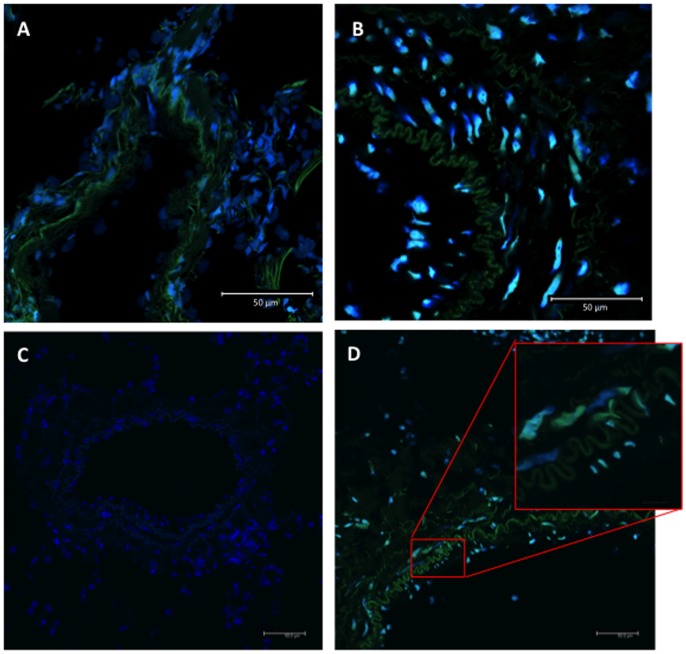
Confocal immunofluorescence NF-κB p65 staining in pulmonary vascular cells. Confocal immunofluorescence staining of acetone-fixed frozen lung specimens using NF-κB KAC310 AlexaFluor488 (cyan-green), and DAPI (blue) counterstaining, showed little positive nuclear staining in control lung specimens (A), and marked nuclear staining in idiopathic PAH (B and D). Antibody negative control staining of a control specimen is shown in C. Some green autofluorescence is seen with collagen fibers. Magnification x400 (insert x2400).

Further confocal immunofluorescence staining experiments demonstrated an intense increase in nuclear p65 staining in pulmonary vascular cells compared to cells from control lung specimens (Figure 7a). These were PASMC and endothelial cells, as identified either side of the elastic tunica media (Figure 7b and 7d). Antibody negative control staining is shown in Figure 7c.

### Polymerase Chain Reaction of Whole Lung Homogenates

**Figure 8:**
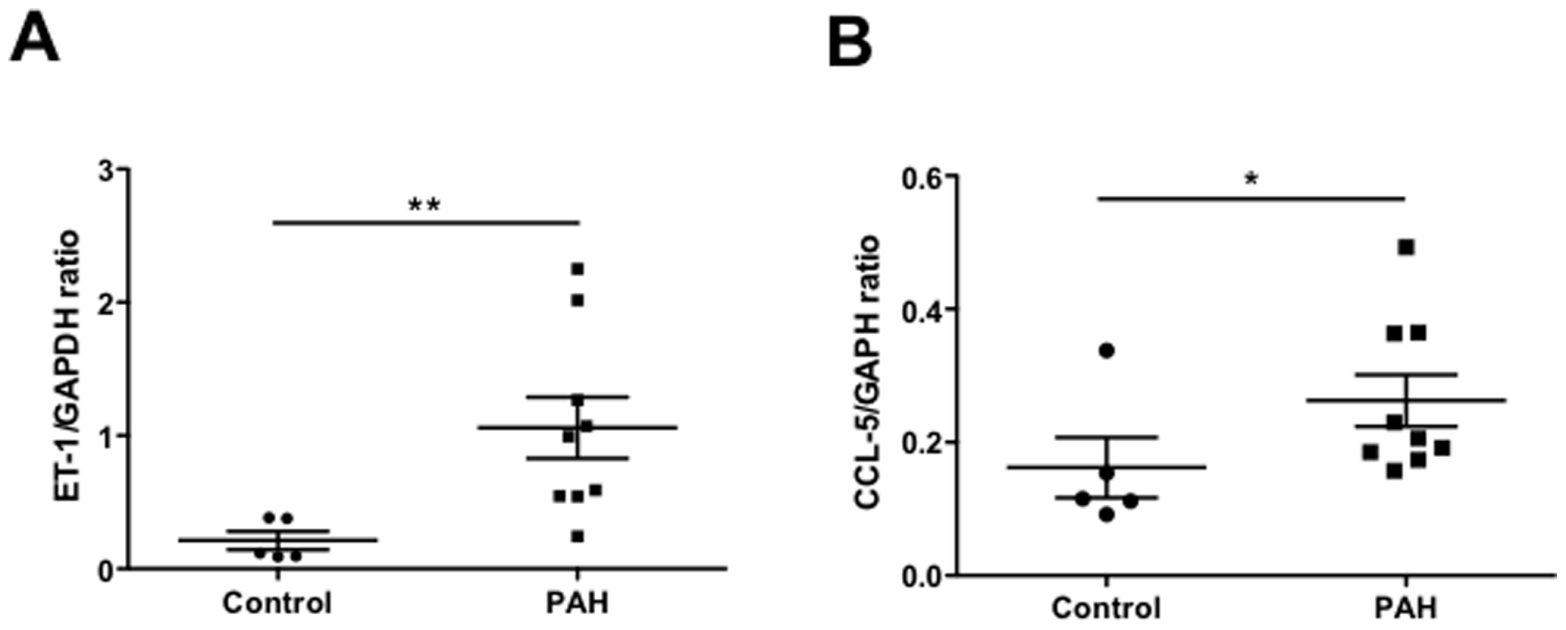
Chemokine analysis in lung tissue. qRT-PCR analysis performed on whole lung homogenates showed an increase in endothelin (ET)-1 mRNA (0.213±0.069 vs. 1.06±0.23, p<0.01) (A) and in CCL5 (RANTES) mRNA (0.16±0.045 vs. 0.26±0.039, p<0.05) (B) in PAH patients vs. controls. Data represent mean±SEM for n = 5 controls and n = 9 PAH patients, and were compared using the Student t test. * p<0.05 ** p<0.01.

Lung sections obtained following transplantation for PAH expressed significantly greater levels of ET-1 and CCL5 mRNA than samples from control subjects undergoing lung resection for pulmonary nodules (Figure 8).

## Discussion

This observational study demonstrates increased NF-κB activation in lung tissue macrophages, lymphocytes, pulmonary arterial endothelial and smooth muscle cells in human idiopathic PAH compared to controls. NF-κB activation was accompanied by an increase in absolute numbers of lung parenchymal macrophages. These findings provide novel data to support a role for NF-κB activation in cells relevant to vascular remodelling in PAH. Furthermore, this work adds to the ever-increasing evidence for a role for inflammation in the pathogenesis of PAH.

There is an increasing literature supporting a role for inflammation in the pathogenesis of PAH [3]–[6]. Perivascular inflammatory infiltrates are prominent in histological specimens [7]–[11], with recent studies suggesting involvement of both the innate [37] and the adaptive immune systems [8]. Circulating cytokines and chemokines are elevated in the plasma of patients with idiopathic PAH [12]–[16] and confer worse prognostic outcome [15], [16]. Activation of NF-κB is central to many chronic inflammatory disorders [21]–[23] [24], [25]. Despite this, there have previously been no reports of NF-κB activation in relevant inflammatory and vascular cells in PAH lung tissue. Although observational in nature we believe the results demonstrating increased NF-κB activation are important. They provide the rationale to consider therapy directed at NF-κB signalling as a potential treatment option. Indeed, there have been several animal models indicating that this rationale may be correct. In the rat monocrotaline (MCT) model of PH, NF-κB activation was increased in rats treated with MCT compared to controls [32], [38], and was associated with loss of EC integrity [32]. NF-κB blockade using the antioxidant pyrrolidine dithiocarbamate at early stages in this model prevented pulmonary vascular remodelling and PH by invasive haemodynamic assessment [32] and reduced macrophage infiltration [38]; Reversal of PH was demonstrated using therapeutic treatment with a nanoparticle-mediated NF-κB oligonucleotide decoy NF-κB inhibitor at 3 weeks after MCT insult after the establishment of PH [31]. Furthermore we have recently shown that dexamethasone, a powerful inhibitor of NF-κB, reversed haemodynamic abnormalities in the MCT model and was associated with improved survival [39].

Endothelial cell p65 nuclear staining was significantly increased in all these patients with end-stage disease, implying a persistent stimulus for inflammation and cell activation throughout the disease process and not just at initiation of endothelial damage, as has been previously suggested [32]. Importantly, we were able to demonstrate elevated levels of ET-1 and CCL5 (RANTES) mRNA in whole lung homogenate of patients with idiopathic PAH compared to controls. Both these mediators have been reported as important in the pathogenesis of idiopathic PAH [17], [40] and are regulated by NF-κB signalling [30]. Relevant activators of NF-κB include oxidants [41], viruses [42], bacterial endotoxin [43], thrombin [44], alveolar hypoxia [45] and abnormal vascular flow [46] as well as positive feedback loops from NF-κB dependent cytokines such as IL-1β and TNF-α [19]. Other downstream gene products of NF-κB activation including MCP-1, CX3CL1 and IL-6 have also been reported to be overexpressed in endothelial cells of PAH patients by immunohistochemistry [47]–[49].

An increase in NF-κB activation was also demonstrated in PASMC in diseased pulmonary arteries and arterioles, although less pronounced compared to ECs. Apart from their obvious role in vessel wall remodelling, PASMC are implicated in the synthesis of vasoactive mediators, growth factors and cytokines. Furthermore, we have demonstrated previously that human PASMC treated *in vitro* with TNF-α and IFN-γ release ET-1 in a synergistic manner due to enhanced NF-κB binding and histone acetylation at specific κB sites [30]. Further direct evidence for a role of NF-κB signalling in human PASMC was demonstrated in a study that associated a single nucleotide polymorphism in the TRPC gene promoter region with a functional NF-κB binding site in 268 patients with idiopathic PAH. PASMC from these patients showed that activation of NF-κB upregulated TRPC6 expression and increased PASMC proliferation; this was attenuated following inhibition of NF-κB [50]. In our study, both EC and PASMC p65+ correlated significantly with vessel medial thickness, suggesting a role in pathogenesis. However, whether NF-κB activation is a primary event in these cells or a consequence of remodelling is not yet determined.

Despite these histological observations, there were few significant correlations with either morphological PH severity (assessed by medial thickness) or clinical markers of disease severity. The only correlations were with macrophage numbers and BNP and SvO_2_, which is interesting and suggests that greater macrophage numbers reflect worse cardiac function. However, no other relationship was found. Reasons for this could relate to the fact that the sample only represented patients with end stage disease: if patients with a spectrum of disease stages were included in the study, correlations may be seen.

Interestingly, in the 2 subjects in our study with known *BMPR2* mutations we observed the most widespread and most intense nuclear p65 staining. Further studies are required to confirm this preliminary observation. BMPR-II dysfunction is an important contributor to remodeling in PAH, both in patients with and without germline mutations [51]. Davies *et al* recently demonstrated increased constitutive nuclear phosphorylation of p65 in PASMC from patients with heritable PAH (harboring *BMPR2* mutations) *in vitro* compared to control PASMC. They suggested that inappropriate NF-κB activation in PASMC from patients with heritable PAH may contribute to enhanced IL-6 and CXCL8 release [29]. Of these, IL-6 is a cytokine associated with worse outcome in IPAH [15], [16] and known to promote vascular remodelling [52]–[56]. In support of this, we also showed that the increase in IL-6 and reduction in *BMPR2* observed in the rat MCT model of PH was reversed by administration of dexamethasone [39].

In this study we showed an increase in the absolute number of lung tissue macrophages. This finding is supported by a recent histological study, which also demonstrated an increase in perivascular monocytes and dendritic cells [10]. Furthermore, we demonstrated increased NF-κB activation within these macrophages. This observation in lung tissue is novel, and adds to the finding that alveolar macrophages from PAH patients obtained at bronchoscopy showed increased NF-κB activation [33], while circulating monocytes showed evidence of immune hyporesponsiveness [57]. Together these findings add to an evolving story where inflammatory cell subsets including dendritic cells [58], [59] and lymphocytes [8] also show lung tissue activation but reduced activation or desensitization in the circulating cell population, suggesting that recruitment of activated inflammatory cells is occurring to the lungs in PAH. Activation of macrophages leads to release of cytokines, chemotaxins, reactive oxygen species and vasoactive mediators, including ET-1 [60], all of which are implicated in the pathogenesis of PAH. Indeed, perivascular macrophages have been shown to stain avidly for ET-1 in histological sections from patients with PAH [40], [61]. Furthermore, macrophages from mice with mutated *BMPR2*, and from humans with heritable PAH (HPAH), were shown to have reduced ET_A_/ET_B_ receptor gene expression, and greater ET-1 release. Finally, stimulation of macrophages in vitro with ET-1 activates NF-κB suggesting a positive feedback loop [61]. Clearly, the role of the macrophage in PAH, and the consequence of its increased NF-κB activation, requires further assessment.

The chief limitation of this study is its observational nature. There are also several other limitations. Firstly, the age between groups, determined by differences in age of presentation of PAH and lung cancer, was markedly different. However, there was no correlation with NF-κB activation and age in our cohort. Increasing age has even been shown to increase NF-κB activation and cytokine release from vascular endothelial cells [62], which if anything would increase the significance of our results. Secondly, comparisons were limited by a lack of invasive hemodynamic data in controls. Although the clinical notes were not examined it is highly unlikely that these patients undergoing lung resection would have significant PAH, as it would be a contra-indication to such surgery. Thirdly, all the patients had a similar level of advanced disease and were on advanced therapies that included prostanoids, phosphodiesterase inhibitors and endothelin receptor antagonists. These medications may of course influence NF-κB signaling although the majority of *in vitro* evidence would suggest down regulation. For instance, treprostinil blocks nuclear translocation of NF-κB and cytokine release [63] and nitric oxide blocks reduction of the NF-κB inhibitory protein IκB-α from LPS-stimulated alveolar macrophages [33]. However, it is clear from our data that in real life, despite advanced therapies, there was still evidence of increased NF-κB activation. The failure to detect a correlation between p65 staining and clinical and haemodynamic indices may reflect the similarity of disease status of the patients and analysis of NF-κB activation in patients with milder disease will be informative.

## Conclusions

In conclusion, we provide novel observations by direct examination of lung tissue that NF-κB is activated in pulmonary macrophages, lymphocytes and pulmonary vascular cells in patients with end stage idiopathic PAH. Although observational in nature, these results support previous animal work suggesting that therapies directed at NF-κB signalling may provide a novel therapeutic option in the future.

## Supporting Information

File S1 **Supplemental data.**
(DOC)
