# Supplementary material for: Nuclear Factor κ-B Is Activated in the Pulmonary Vessels of Patients with End-Stage Idiopathic Pulmonary Arterial Hypertension
Source: PLoS One. 2013 Oct 4;8(10):e75415. doi: 10.1371/journal.pone.0075415 (PMC3790752; doi:10.1371/journal.pone.0075415)
Supplement: File S1 — Supplemental data. (DOC) [file pone.0075415.s001.doc]

**Supplemental Figures S1-5**

Figure S1 No correlations seen with pulmonary arterial medial thickness and clinical parameters in patients with PAH (pulmonary arterial hypertension)

Morphometric analysis was performed to assess mean pulmonary arterial medial thickness (percentage vessel thickness / external diameter) of haematoxylin & eosin stained of lung sections, by 2 independent blinded observers. Correlations were made with clinical parameters including 6-minute walk distance (A); brain natriuretic peptide (BNP) (B); oxygen saturations in the central vein (SvO_2_) (C); mean pulmonary arterial pressure (mPAP) (D); cardiac index (E) and right atrial pressure (F). Data represent Pearson’s correlation co-efficient (r) for up to 12 patients per group.


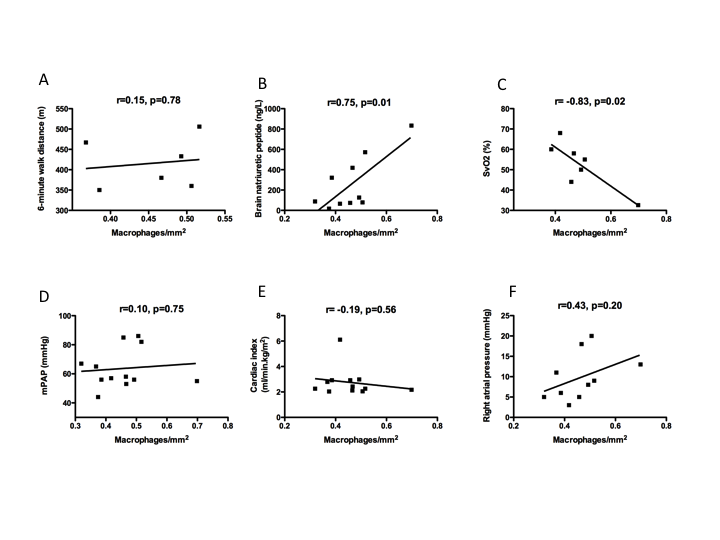


Figure S2: Correlations seen with macrophage counts and clinical parameters in patients with PAH (pulmonary arterial hypertension)

Morphometric analysis was performed to assess macrophage counts on lung sections from PAH patients double-immunostained for CD68 and p65, with haematoxylin & eosin counterstaining, by 2 independent blinded observers. Correlations were made with clinical parameters including 6-minute walk distance (A); brain natriuretic peptide (BNP) (B); oxygen saturations in the central vein (SvO_2_) (C); mean pulmonary arterial pressure (mPAP) (D); cardiac index (E) and right atrial pressure (F). Correlations were significant only for BNP and SvO_2_. Data represent Pearson’s correlation co-efficient (r) for up to 12 patients per group.


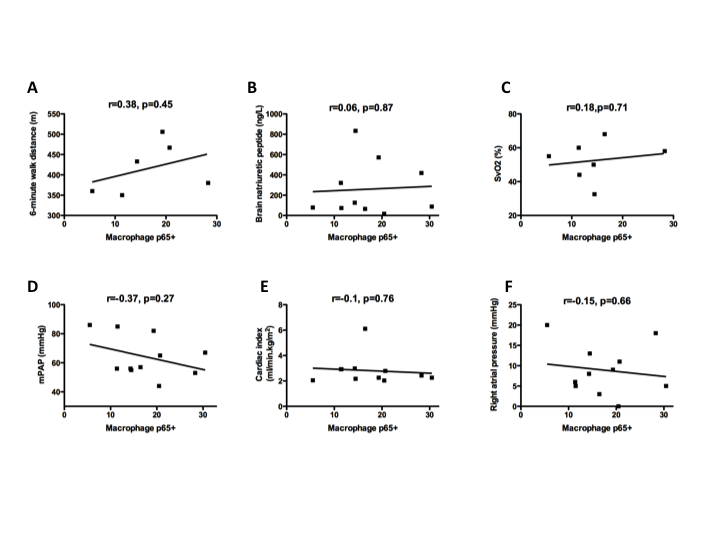


Figure S3: No correlation of macrophage nuclear positivity (p65+) and clinical parameters in patients with pulmonary arterial hypertension (PAH)

Morphometric analysis was performed to assess macrophage counts on lung sections from PAH patients double-immunostained for CD68 and p65, with haematoxylin & eosin counterstaining, by 2 independent blinded observers. Correlations were made with clinical parameters including 6-minute walk distance (A); brain natriuretic peptide (BNP) (B); oxygen saturations in the central vein (SvO_2_) (C); mean pulmonary arterial pressure (mPAP) (D); cardiac index (E) and right atrial pressure (F). No correlation was found for macrophage nuclear p65+ with any of the recorded clinical parameters in the PAH patient group. Data represent Pearson’s correlation co-efficient (r) for up to 12 patients per group.


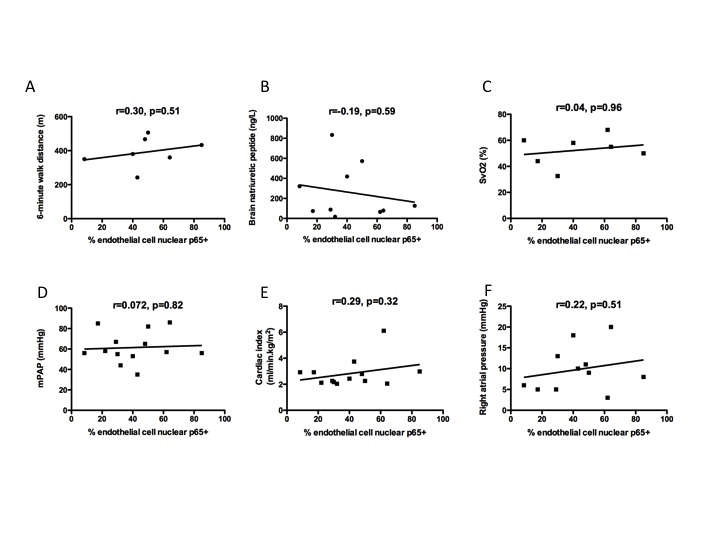


Figure S4: No correlation of endothelial cell nuclear p65 positivity (p65+) with clinical severity parameters in patients with pulmonary arterial hypertension (PAH)

No significant correlation was found for endothelial cell p65+ with any of the recorded clinical parameters in the PAH patient group including 6-minute walk distance (A); BNP (B); SvO_2_ (C); mean pulmonary arterial pressure (D); cardiac index (E); and right atrial pressure (F). Solid squares points are PAH patients. Data represent Pearson’s correlation co-efficient (r) for up to 14 patients per group.


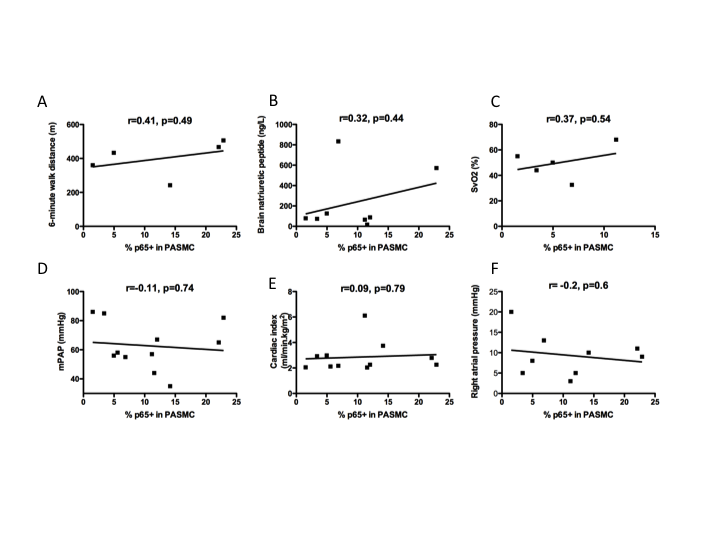


Figure S5: No correlation of PASMC p65 positivity (p65+) with clinical severity parameters in patients with pulmonary arterial hypertension (PAH)

No significant correlation was found for PASMC p65+ with any of the recorded clinical parameters in the PAH patient group including 6-minute walk distance (A); BNP (B); SvO_2_ (C); mean pulmonary arterial pressure (D); cardiac index (E); and right atrial pressure (F). Solid squares points are PAH patients. Data represent Pearson’s correlation co-efficient (r) for up to 14 patients per group.
